# Night Air

**Published:** 1881-11

**Authors:** 


### NIGHT AIR.

Before we can hope to fight consumption with any chance of success, we have to get rid of the *night air superstition.* Like the dread of cold water, raw fruit, etc., it is founded on mistrust of our instincts. It is probably the most prolific single cause of impaired health, even among the civilized nations of our enlightened age, though its absurdity rivals the grossest delusions of the witchcraft era. The subjection of holy reason to hearsays could hardly go further. "Beware of the night-wind; be sure and close your windows after dark!" In other words beware of God's free air; be sure and infect your lungs with the stagnant, azotized, and offensive atmosphere of your bedroom. In other words, beware of the rock spring; stick to sewerage. Is night air injurous? Since the day of creation, that air has been breathed with impunity by millions of different animals—tender, delicate creatures, some of them—fawns, lambs and young birds. The moist night air of the tropical forests is breathed with impunity by our next relatives, the anthropoid apes—the same apes that soon perish with consumption in the close though generally well-warmed atmosphere of our northern menageries. Thousands of our soldiers, hunters, and lumbermen, sleep every night in tents and open sheds without the least injurious consequences; men in the last stage of consumption have recovered by adopting a semi-savage mode of life, and camping out-doors in all but the stormiest nights. Is it the draught you fear, or the contrast of temperature? Blacksmiths and railroad conductors seem to thrive under such influences. Draught? Have you never seen boys skating in the teeth of a snow-storm at the rate of fifteen miles an hour? "They counteract the effect of the cold air by vigorous exercise." Is there no other way of keeping warm? Does the north wind damage the fine lady sitting motionless in her sleigh, or the helmsman of a storm-tossed vessel? It cannot be the *inclemency* of the open air, for, even in sweltering summer nights, the sweet south wind, blessed by all creatures that draw the breath of life, brings no relief to the victim of aerophobia. There is no doubt that families who have freed themselves from the curse of that superstition can live out and out healthier in the heart of a great city than its slaves on the airiest highland of the southern Appenines. *Popular Science Monthly.*

